# Distress among cancer patients attending rehabilitation in the community

**DOI:** 10.1007/s00520-021-06422-3

**Published:** 2021-07-17

**Authors:** Ann Kuo

**Affiliations:** Cancer Rehabilitation Services, Singapore Cancer Society, Singapore, Singapore

**Keywords:** Distress, Cancer rehabilitation, Community, Age, Symptom clusters

## Abstract

**Purpose:**

The aim of this study is to identify sources of distress among cancer patients attending rehabilitation in the community.

**Methods:**

Participants were 430 patients recruited from a cancer rehabilitation center in Singapore between 2017 and 2018, who had rated their distress using the distress thermometer (DT) and indicated associated problems on the problem list. Chi-square tests were used to detect differences in the reported symptoms among three age groups. Exploratory factor analysis was used to identify symptom clusters. Partial correlational analysis was then performed to examine the relationship between distress, symptom clusters, and age controlling for gender and cancer type.

**Results:**

About 30% of the participants reported distress ≥ 5 on the DT (mean 3.3 ± 2.5), and the mean number of problems endorsed was 8 ± 6. A higher total number of reported problems (*r* = .63) and younger age (*r* =  − .21) were associated with increased distress. The younger age group also reported more problems surrounding emotions, finance, work/school, children-related issues, and physical symptoms such as sleep and nausea. Of the 12 factors identified, 9 psychosocial and physical symptom clusters correlated with distress (*r* ranging from .12 to .41). All results were statistically significant after adjustment (*p* ≤ 0.05).

**Conclusion:**

Younger survivors are more at risk of distress and report greater role functioning concerns related to childcare, partner relationship, and work participation. Age-tailored and multimodal interventions may be necessary to adequately address age-related differences and help coordinate management of multiple symptom clusters across physical and psychosocial concerns.

## Introduction

Although distress level has been found to decline with time, unmet psychosocial needs and physical symptoms may emerge along the cancer trajectory (such as treatment-induced side effects, recurrence, and advanced disease) and lead to enduring distress among cancer survivors [1, 2]. Continual elevated level of distress has also been reported in cancer survivors 5 years post-cancer treatment and diagnosis, suggesting that distress and its associated problems may evolve or remain unresolved, requiring further intervention [3]. Conducting distress screening in the community is, therefore, necessary to help identify distressed cancer survivors for treatment through appropriate referrals to services and by providing effective management strategies to address underlying concerns [4–6].

The National Comprehensive Cancer Network’s Distress Thermometer (DT) is developed to screen for distress, and a cut-off score has been used to indicate the need to intervene, informed by a 39-item Problem List (PL) [7]. Optimal cut-off score varies across cultures, clinical settings, cancer stages, and types; a cut-off score of 4 or 5 on the DT is generally recommended [8–10]. The accompanied PL items have been used to ascertain key concerns that may have perpetuated distress. Problems that most contributed to distress include emotional issues (particularly worry) and insurance/financial challenges which evidenced the need to provide emotional and financial counseling; the more commonly endorsed physical symptoms associated with distress are fatigue and sleep, indicating necessity for education and further rehabilitation [10–12].

Distress and symptom experiences have been observed to vary by age group [13–15]. Younger survivors are more likely to report higher distress and more depressive symptoms than older survivors [16, 17]. In addition, patients who are younger may indicate more pain, sexual, and work/school issues, while older patients may present with nutrition, shortness of breath, and mobility concerns [14, 18, 19]. In a sample of patients undergoing active cancer treatment, the percentage of patients reporting common symptoms like worry, fatigue, sleep, and pain was also found to be significantly lower in the older age group [19]. Age-related differences in distress rating, the type, and frequency of problems reported may help to underscore the importance of attending to disparate needs of cancer survivors across the life span.

Some of the cancer-related symptoms co-occur and have the potential to interact, leading to greater distress [14, 20]. Symptom clusters that consist of two or more related concurrent symptoms may arise due to the disease, its treatment, or exacerbation from a triggering symptom [21]. Some of the symptom clusters include the psychoneurological cluster comprising of insomnia, fatigue, depression, pain, and cognitive disturbance; the gastrointestinal cluster involving nausea and vomiting; and the emotional cluster encompassing anxiety and depression [22, 23]. Identifying concurrent symptoms and prioritizing strategies to address possible underlying mechanism may help reduce distress and simplify management of multiple problems [20].

At present, the types of problems faced by cancer patients attending rehabilitation in the community and their relationships to distress are not well-established. Specifically, the extent to which age-related differences and symptom clusters affect these cancer survivors remains uncertain. This study aims to identify sources of distress using the problem list endorsed by cancer patients and examine the impact of age and symptom clusters on distress rating for the purpose of recommending appropriate intervention strategies and management of distress in community cancer rehabilitation.

## Methods

The study design was a retrospective analysis of cross-sectional baseline data collected between 2017 and 2018 at a cancer rehabilitation center based in a community outpatient setting in Singapore. Exemption from ethics approval was granted by the institutional review board from the Agency for Integrated Care. All participants had consented to the collection and use of their data for research and development purposes, in compliance with the Singapore Personal Data Protection Act 2012, prior to completing the DT and the PL.

### Participants

Research participants were 430 cancer patients, predominantly female and with breast cancer. Approximately 30% of the cancer patients reported a distress level of 5 or greater on the DT, and the mean number of problems reported on the PL was 8 ± 6 for the total sample; 10 ± 6 for patients ≤ 54 years of age; 8 ± 6 for patients between 55 and 64 years of age; and 7 ± 6 for patients ≥ 65 years of age. Characteristics of the participants are shown in Table 1. Patients with cognitive or severe visual and hearing impairment were excluded. The majority of the participants were able to complete the self-administered questionnaire with minimal or no assistance.

**Table 1:** Characteristics of cancer patients (*n* = 430)

Variable	Range	Mean ± SD	
Age	28–90	60.0 ± 11.1	
Distress (total sample)	0–10	3.3 ± 2.5	
≤ 54 years (*n* = 134)		4.0 ± 2.5	
55–64 years (*n* = 137)		3.2 ± 2.6	
≥ 65 years (*n* = 152)		2.7 ± 2.4	
Low distress	0–4	1.9 ± 1.4	
High distress	5–10	6.5 ± 1.4	
Number of problems endorsed	0–32	8 ± 6	
Variable	*N*	Percentage	
Gender			
Female	291	67.7	
Male	139	32.3	
Cancer type			
Breast	200	46.5	
Prostate	80	18.6	
Colorectal	43	10.0	
Gynecologic	25	5.8	
Lung	21	4.9	
Blood	16	3.7	
Head and neck	14	3.3	
Others	31	7.2	
Distress rating			
≥ 4	175	40.7	
≥ 5	132	30.7	

### Procedure

The DT and the PL were administered during initial admission into the cancer rehabilitation program as part of routine screening. The participants completed the questionnaire in the presence of a staff to answer any questions, and assistance was provided for participants who were illiterate.

### Instrument

Distress was measured by the DT that required participants to indicate their level of distress experienced in the past week including today from 0 (no distress) to 10 (extreme distress). The validity of the DT for distress screening using the Singapore cancer population was established; a cut-off score of 5 on the DT was shown to have the best sensitivity (0.88) and specificity (0.81) [10].

Distress symptoms were determined using the PL with the participants answering yes or no to a list of 39 items related to practical, family, emotional, spiritual/religious, and physical problems that they encountered within the past week including today. Internal consistency was found to be adequate using this study sample (Cronbach’s alpha = 0.88).

### Statistical analysis

Descriptive analysis was employed to compare the frequency of commonly reported problems among three groups of cancer patients (≤ 54 years of age; 55–64 years of age; and ≥ 65 years of age). Chi-square tests were used to assess differences in the proportions of various reported problems across three age groups. The Spearman’s rank-order correlation analysis was also conducted to examine the relationship between distress rating and the problems/symptoms reported.

Appropriateness of the data for exploratory factor analysis (EFA) was determined by the Kaiser–Meyer–Olkin measure of sampling adequacy (KMO > 0.60) and a significance of α < 0.05 on the Bartlett’s test of sphericity. The number of factors to be extracted was based on an eigenvalue > 1, the scree plot, and attaining a total variance of approximately 60%. Items on the PL that did not load well on any of the factors (factor loading < 0.30) and with low communalities (< 0.40) would be removed. Principal component analysis with orthogonal rotation (equamax) was performed to help simplify interpretation of the symptom factors. Factor scores were generated using regression method; bivariate and partial correlation analyses were conducted to further examine the relationship among symptom factors/clusters, distress, total number of items endorsed on the PL, and age, controlling for gender and cancer type.

All analyses were performed using IBM SPSS Statistics for Windows, Version 26.0 (Armonk, NY: IBM Corp) and conducted at the 0.05 significance level.

## Results

Table 2 shows the relationship between distress and the reported symptoms ranked by the most distressing symptoms from emotions, loss of interest in usual activities, finance, and work or school concerns to change in urination. Among the physical symptoms, fatigue, sleep, difficulty with bathing and dressing, memory, and breathing issues were more associated with distress. Age-related differences were also revealed with the younger age group (≤ 54 years of age) reporting more problems surrounding emotions, finance, work/school, children-related issues, and physical symptoms such as sleep and nausea. Change in urination was more frequently reported by the oldest age group (≥ 65 years of age).

**Table 2:** Correlates of distress and the differences in frequency of reported symptoms across age groups

Symptoms	*N* (%)	DT^a^	Age group			*p* value^b^
			≤ 54 (%)	55–64 (%)	≥ 65 (%)	
Worry	224 (53.0)	.49**	92	70	62	< .001
Sadness	130 (30.7)	.47**	54	42	34	< .01
Fears	149 (35.2)	.46**	70	42	37	< .001
Depression	99 (23.4)	.44**	41	29	29	ns
Nervousness	115 (27.2)	.43**	46	33	36	ns
Loss of interest	89 (21.0)	.39**	29	32	28	ns
Finance	93 (22.0)	.33**	39	31	23	< .05
Fatigue	226 (53.4)	.32**	83	67	76	ns
Work/school	70 (16.5)	.32**	43	22	5	< .001
Sleep	196 (46.3)	.30**	76	57	63	< .05
Transportation	48 (11.3)	.27**	17	11	20	ns
Housing	43 (10.2)	.26**	19	16	8	< .05
Bath/dress	36 (8.5)	.26**	15	11	10	ns
Deal children	34 (8.0)	.26**	17	13	4	< .01
Treatment decision	73 (17.3)	.25**	28	27	18	ns
Appearance	64 (15.1)	.25**	27	19	18	ns
Memory	178 (42.1)	.24**	63	60	55	ns
Family health	77 (18.2)	.24**	29	31	17	< .05
Breathing	75 (17.7)	.24**	19	26	30	ns
Eating	69 (16.3)	.24**	27	21	21	ns
Deal partner	52 (12.3)	.24**	19	18	15	ns
Nausea	42 (9.9)	.23**	20	13	9	< .05
Indigestion	64 (15.1)	.22**	24	25	15	ns
Pain	180 (42.6)	.21**	57	63	60	ns
Fevers	25 (5.9)	.20**	11	7	7	ns
Spiritual	18 (4.3)	.20**	9	7	2	ns
Nose dry/congest	65 (15.4)	.19**	25	19	21	ns
Tingling	191 (45.2)	.18**	65	65	61	ns
Get around	61 (14.4)	.17**	22	14	25	ns
Feel swollen	96 (22.7)	.16**	39	24	33	ns
Mouth sore	26 (6.1)	.15**	9	12	5	ns
Skin dry/itch	169 (40.0)	.14**	52	54	63	ns
Constipation	76 (18.0)	.14**	29	22	25	ns
Diarrhea	36 (8.5)	.14**	14	9	13	ns
Sexual	34 (8.0)	.13**	10	15	9	ns
Substance abuse	6(1.4)	.12*	2	1	3	ns
Childcare	25 (5.9)	.10*	13	7	5	ns
Ability to have child	3 (0.7)	.08	3	0	0	< 0.05
Change in urination	55 (13.0)	.06	6	17	32	< .001

Note: *DT*, distress thermometer; *ns*, not significant at *p* < .05 level. *p < .05. **p < .01

^a^Spearman’s rank-order correlation

^b^Chi-squared test

Exploratory factor analysis yielded 12 factors accounting for 59.9% of the total variance, presented in Table 3. Kaiser–Meyer–Olkin measure of sampling adequacy was found to be adequate at 0.85, and the Bartlett’s test of sphericity was also significant at *p* < 0.001. Thirty-six PL items were retained. Distinct symptom clusters and one factor identified were (1) emotional (nervousness, fears, depression, and worry); (2) gastrointestinal (nausea, fevers, indigestion, and diarrhea); (3) fatigue (fatigue, sleep, and memory); (4) financial (housing, insurance/finance, and family health issues); (5) sensory (tingling, pain, and skin itch); (6) functional ability (bathing/dressing and getting around); (7) respiratory (breathing and nose congested); (8) relationship (deal with partner and children); (9) appearance (appearance and treatment decisions); (10) constipation (constipation, swelling, and mouth sores); (11) genitourinary (change in urination and sexual problems); and (12) childcare.

**Table 3:** Symptom factors with factor loadings and communalities based on exploratory factor analysis

Items	Communalities	Factor loadings
Factor 1: emotional		
Depression	.64	.73
Sadness	.69	.66
Fears	.66	.61
Nervousness	.58	.60
Loss of interest	.53	.54
Worry	.58	.47
Factor 2: gastrointestinal		
Nausea	.60	.67
Transportation	.57	.65
Fevers	.54	.59
Indigestion	.59	.59
Diarrhea	.53	.39
Factor 3: fatigue		
Fatigue	.57	.60
Sleep	.56	.58
Memory	.53	.55
Work/school	.67	.49
Factor 4: finances		
Housing	.68	.76
Insurance/financial	.63	.66
Family health issues	.47	.49
Factor 5: sensory		
Tingling	.55	.68
Pain	.57	.67
Skin dry/itch	.50	.51
Factor 6: functional ability		
Bath/dress	.71	.81
Get around	.67	.76
Factor 7: respiratory		
Breathing	.65	.77
Nose dry/congest	.64	.59
Eating	.55	.44
Factor 8: relationship		
Deal with partner	.68	.75
Deal with children	.63	.72
Factor 9: appearance		
Appearance	.65	.77
Treatment decisions	.47	.34
Factor 10: constipation		
Constipation	.53	.67
Feeling swollen	.53	.47
Mouth sores	.65	.42
Factor 11: genitourinary		
Change in urination	.71	.79
Sexual problems	.67	.62
Factor 12: childcare	.60	.73

The zero-order and partial correlation coefficients for distress rating, age, and symptom clusters/factors are detailed in Table 4. A higher total number of reported problems (*r* = 0.63, *p* < 0.01) and younger age (*r* =  − 0.21, *p* < 0.01) were associated with increased distress after controlling for gender and cancer type. Younger patients are also likely to endorse more problems (*r* =  − 0.22, *p* < 0.01). All symptom factors except sensory, constipation, and childcare correlated with distress, even after controlling for gender and cancer type. Emotional symptom cluster was the most distressing followed by financial, appearance, relationship, fatigue, functional ability, gastrointestinal, respiratory, and genitourinary symptom clusters. Fatigue, financial, appearance, and childcare correlated with age.

**Table 4:** Relationship among distress, age, and symptom clusters/factors, adjusting for gender and cancer type (*N* = 430)

Variable	DT		Age	
	Zero-order	Partial	Zero-order	Partial
Emotional	.40**	.41**	− .10	− .09
Gastrointestinal	.17**	.16**	− .03	− .03
Fatigue	.22**	.21**	− .16**	− .14*
Financial	.24**	.25**	− .14**	− .16**
Sensory	.07	.06	− .04	− .05
Functional ability	.20**	.20**	.03	.03
Respiratory	.15**	.15**	− .03	− .03
Relationship	.22**	.21**	− .10	− .08
Appearance	.23**	.23**	− .24**	− .25**
Constipation	.00	0.01	.01	.04
Genitourinary	.08	.12*	.16**	.08
Childcare	.07	− .07	− .17**	− .15**

Note: *DT*, distress thermometer. *PL*, problem list, *p < .05. **p < .01

## Discussion

Findings from this study suggest that between thirty and forty percent of cancer patients attending rehabilitation in the community may be at risk for distress, and these patients may often report multiple psychosocial and physical problems concurrently. The rate of distress among cancer patients in this sample appears comparable to prior studies that estimated between 25 to 40% of cancer survivors experiencing continual level of elevated distress post-cancer treatment [3, 24]. In particular, the rate of distress using a cut-off score of ≥ 5 on the DT is close to reported rates from the Netherlands using study samples of survivors with breast cancer measured at 12 to 15-month post-diagnosis [2, 3]. The mean number of problems endorsed also parallels another study that reported 7.62 ± 5.75 mean items endorsed by cancer patients at risk for distress [4]. The rate of types of problems endorsed, sampled from a cancer rehabilitation clinic in Sweden, is similarly high for worry, fatigue, sleep, memory, pain, skin dryness/itch, and tingling [25]. Further, among the common problems reported by cancer survivors, emotional concerns, loss of interest in usual activities, finance, fatigue, work/school, and sleep may be the most distressing, evidencing the need for a holistic approach in cancer rehabilitation to deal with a mixed array of psychosocial and physical problems [26].

Younger cancer patients may be more at risk of distress, presenting with a greater mean distress rating and higher total number of reported problems compared to older cancer patients. Salient age-related psychosocial problems appear to surround (1) negative emotions; (2) employment and finances; (3) appearance; and (4) family relationships especially in dealing with children [27–29]. Having multiple role demands that are specific to a younger age (e.g., parenting young children, completing education, establishing career and finances, and managing marital relationship) may have contributed to greater distress [28]. As this study sample consists predominantly of women with breast cancer, some younger survivors may also be more concerned with body image and attractiveness after surgery and mastectomy, further affecting their emotions and the quality of their intimate relationship [30]. In addition, breast cancers in women under 40 years of age tend to be diagnosed at a later stage, presenting with more aggressive features and poorer outcomes, thus potentially leading to heightened distress. [31] Besides providing early support, education, and counseling services to this high-risk younger group, rehabilitation interventions should address the impact of cancer on patients’ role functioning related to family life, partner relationship, and work participation as these problems are more commonly reported in distressed younger survivors [28].

Several symptom clusters are related to distress with the emotional symptom cluster appearing to be the most distressing. Emotional symptom cluster has also been shown to be the most burdensome over time compared to other symptom clusters and significantly attenuate the health status and quality of life of breast cancer survivors [32]. Losing interest in activity is a major hallmark of depression, suggesting that emotional issues may be accompanied by a reduction in activity participation. A prior study reported an average of 12% reduction in activity level especially in high physical demand leisure and social activities among older cancer survivors [33]. Despite performing fewer activities, cancer survivors may prefer to focus their resources on more enjoyable, creative, and meaningful engagements to help alleviate their mood [33]. Activity-focused rehabilitation that engages cancer survivors in a process of goal setting, evaluation, and problem-solving facilitates building of confidence in managing residual impairments [34]. Increased self-efficacy in coping with their symptoms during daily activities may in turn enhance cancer survivors’ emotional well-being [35]. Supporting activity participation may serve as a key strategy to manage the emotional symptom cluster and promote better quality of life in cancer survivors [33, 36, 37].

Co-existing symptoms within a cluster often interact and elevate distress. For instance, having sleep disturbance may increase fatigue, and both sleep disturbance and fatigue affect cognitive function [38, 39]. Further, the presence of fatigue especially mental fatigue and cognitive dysfunction predisposes cancer survivors to having difficulties at work [40, 41]. Educating cancer patients on the relationship among co-occurring symptoms may lead to more effective management of the clustered symptoms and hence alleviate distress [20]. Multidisciplinary interventions combining psychoeducation, physical, and vocational components have demonstrated efficacy in supporting cancer survivors return to their worker role [42]. This study suggests a need to prioritize strategies to address the interaction of sleep, fatigue, and cognitive function within return to work or school interventions. Practical support may also be necessary to help deescalate distress for some symptom clusters. For example, cancer patients with gastrointestinal symptoms may experience a greater burden of distress commuting to clinic for chemotherapy compared to patients without such treatment side effects [43]. The gastrointestinal symptom cluster—commonly characterized by nausea and vomiting—significantly affect cancer patients’ daily function and quality of life [44, 45]; addressing transportation barriers for cancer patients with ongoing treatments may thus be beneficial. Other practical support may include financial assistance. Financial hardship may arise not only from cancer treatment–related out-of-pocket costs, but also reduced income, missed work, and/or medical debt [46]. Findings from this study underscore the need to consider housing and health issues of other family members when assessing financial distress and providing financial aid as the economic impact of cancer often affects the whole family [47].

In simplifying management of symptom clusters, strategies may be prescribed based on their potential benefits in associated symptoms or across multiple symptom clusters [20]. For example, exercise has been shown to improve emotional well-being and concurrently help reduce fatigue and pain and enhance sleep and physical and cognitive function [48–50]. Some studies have attributed the development of symptom cluster to inflammatory mechanisms, and exercise has been found to downregulate pro-inflammatory cytokines and upregulate anti-inflammatory cytokines, hence providing beneficial effects across a wide spectrum of symptoms [51, 52]. Besides exercise, stress management delivered through various intervention strategies such as cognitive behavioral therapy, mindfulness practice, deep breathing, and relaxation training has demonstrated positive effects on emotional well-being, pain, and fatigue [46, 53–55]. To effectively manage distress-related symptoms, multimodal interventions that combine strategies such as exercise, stress management, nutrition, and psychoeducation have evidenced promising results with significant symptom reduction across diverse symptom clusters [56, 57].

Despite some limitations, this study provides insight on the relationship of symptom clusters to distress among cancer survivors to further understand their rehabilitation needs in the community setting. This study is limited by not accounting for socioeconomic status, stage of cancer, and the type of treatment received as these factors are also related to distress [58]. In addition, the problem list items are inadequate to cover issues faced by patients with different types of cancer, items such as swallowing difficulty and dry mouth related to head and neck cancer are not considered. In addition, some problem list items may be ambiguous, such as eating and feeling swollen. Eating may be related to dietary concern, loss of appetite, and/or feeding issue. Patients with poor appetite may not report on eating if they interpret eating as more of a feeding difficulty. Likewise, patients with lymphedema concern are less likely to endorse on the item feeling swollen as feeling swollen may also mean swelling and inflammation or bloating. Future studies should examine (1) the occurrence of symptom clusters based on a refined problem list that include clearly worded items relevant to common cancer types and (2) the relationship among co-existing symptoms for specific age group controlling for socioeconomic status, stage of disease, and treatment received.

In summary, cancer survivors, who are in distress, are more likely to experience multiple symptoms across physical and psychosocial domains. Younger survivors may be more at risk of distress with multiple roles to fulfill. Recognizing concurrent symptoms and providing coordinated rehabilitation strategies to manage across symptom clusters for different age groups are necessary to help reduce enduring distress among cancer survivors in the community.

## Data Availability

N/A

## Code availability

N/A
